# *C9orf72* dipeptides activate the NLRP3 inflammasome

**DOI:** 10.1093/braincomms/fcae282

**Published:** 2024-08-20

**Authors:** Jack Rivers-Auty, Christopher Hoyle, Ayesha Pointer, Catherine Lawrence, Stuart Pickering-Brown, David Brough, Sarah Ryan

**Affiliations:** School of Medicine, University of Tasmania, Hobart, TAS 7000, Australia; Division of Neuroscience, Faculty of Biology, Medicine and Health, School of Biological Sciences, University of Manchester, Manchester M13 9PL, UK; Geoffrey Jefferson Brain Research Centre, Manchester Academic Health Science Centre, Northern Care Alliance NHS Group, University of Manchester, Manchester M13 9PT, UK; Division of Neuroscience, Faculty of Biology, Medicine and Health, School of Biological Sciences, University of Manchester, Manchester M13 9PL, UK; Geoffrey Jefferson Brain Research Centre, Manchester Academic Health Science Centre, Northern Care Alliance NHS Group, University of Manchester, Manchester M13 9PT, UK; Division of Neuroscience, Faculty of Biology, Medicine and Health, School of Biological Sciences, University of Manchester, Manchester M13 9PL, UK; Geoffrey Jefferson Brain Research Centre, Manchester Academic Health Science Centre, Northern Care Alliance NHS Group, University of Manchester, Manchester M13 9PT, UK; Division of Neuroscience, Faculty of Biology, Medicine and Health, School of Biological Sciences, University of Manchester, Manchester M13 9PL, UK; Division of Neuroscience, Faculty of Biology, Medicine and Health, School of Biological Sciences, University of Manchester, Manchester M13 9PL, UK; Geoffrey Jefferson Brain Research Centre, Manchester Academic Health Science Centre, Northern Care Alliance NHS Group, University of Manchester, Manchester M13 9PT, UK; Division of Neuroscience, Faculty of Biology, Medicine and Health, School of Biological Sciences, University of Manchester, Manchester M13 9PL, UK; Geoffrey Jefferson Brain Research Centre, Manchester Academic Health Science Centre, Northern Care Alliance NHS Group, University of Manchester, Manchester M13 9PT, UK

**Keywords:** *c9orf72*, frontotemporal dementia, ALS, NLRP3, inflammasome

## Abstract

Frontotemporal dementia and amyotrophic lateral sclerosis are neurodegenerative diseases with considerable clinical, genetic and pathological overlap. The most common cause of both diseases is a hexanucleotide repeat expansion in *C9orf72*. The expansion is translated to produce five toxic dipeptides, which aggregate in patient brain. Neuroinflammation is a feature of frontotemporal dementia and amyotrophic lateral sclerosis; however, its causes are unknown. The nod-like receptor family, pyrin domain-containing 3 inflammasome is implicated in several other neurodegenerative diseases as a driver of damaging inflammation. The inflammasome is a multi-protein complex which forms in immune cells in response to tissue damage, pathogens or aggregating proteins. Inflammasome activation is observed in models of other neurodegenerative diseases such as Alzheimer’s disease, and inflammasome inhibition rescues cognitive decline in rodent models of Alzheimer’s disease. Here, we show that a dipeptide arising from the *C9orf72* expansion, poly-glycine–arginine, activated the inflammasome in microglia and macrophages, leading to secretion of the pro-inflammatory cytokine, interleukin-1β. Poly-glycine–arginine also activated the inflammasome in organotypic hippocampal slice cultures, and immunofluorescence imaging demonstrated formation of inflammasome specks in response to poly-glycine–arginine. Several clinically available anti-inflammatory drugs rescued poly-glycine–arginine-induced inflammasome activation. These data suggest that *C9orf72* dipeptides contribute to the neuroinflammation observed in patients, and highlight the inflammasome as a potential therapeutic target for frontotemporal dementia and amyotrophic lateral sclerosis.

## Teaser

A C9orf72 repeat expansion causes frontotemporal dementia and amyotrophic lateral sclerosis. The expansion is translated to produce dipeptides that activate the inflammasome in immune cells, causing release of the pro-inflammatory cytokine interleukin-1β. This suggests that C9orf72 dipeptides cause neuroinflammation in disease, and highlights the inflammasome as a therapeutic target.

## Graphical Abstract

**Graphical Abstract:**
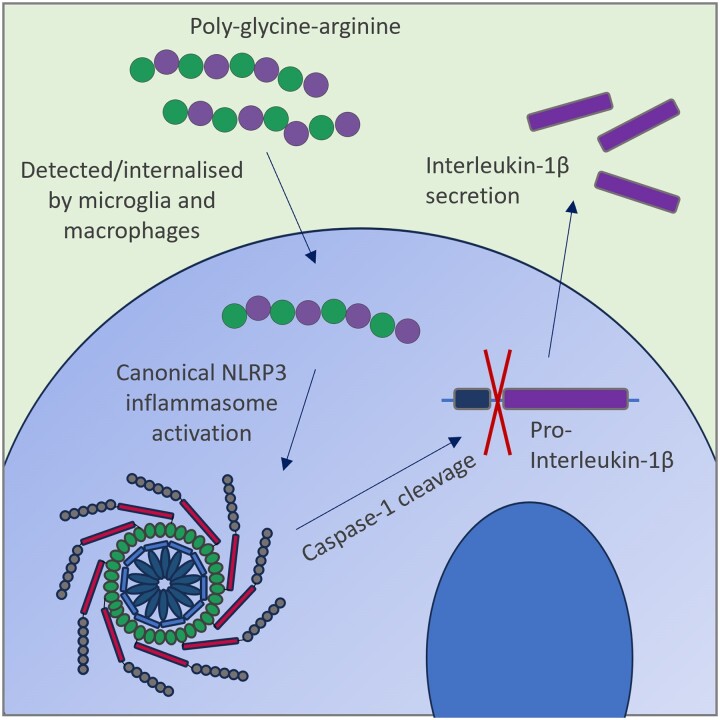

## Introduction

Frontotemporal dementia (FTD) is the second most common cause of young-onset dementia, typically affecting people in their mid to late 50s. Symptoms include language difficulties, personality changes, disinhibition and behavioural changes. Approximately 15% of FTD patients also develop amyotrophic lateral sclerosis (ALS), a devastating neurodegenerative disease causing progressive paralysis and which is almost invariably fatal within 2–5 years of symptomatic onset. There are no effective treatments currently available for either disease. The most common cause of both FTD and ALS is a hexanucleotide repeat expansion in *C9orf72*^1,2^ (C9FTD/ALS). The expansion is unconventionally translated via repeat-associated non-ATG translation to produce five dipeptide repeat proteins (DPRs): glycine–alanine (GA), glycine–arginine (GR), proline–arginine (PR), alanine–proline (AP) and glycine–proline (GP). These DPRs form insoluble intracellular inclusions in patient brain.^3,4^ DPRs are highly toxic in multiple animal and cell culture models, with GR widely considered as the most severely toxic DPR^5-10^ However, the mechanisms underlying DPR toxicity remain unclear.

Neuroinflammation is a common hallmark of all neurodegenerative diseases, including FTD and ALS. Immunohistochemical analysis of FTD/ALS patient brain tissue and PET imaging of living patients show increased microglial activation in affected brain regions.^11,12^ Furthermore, elevated levels of pro-inflammatory cytokines are observed in FTD/ALS patient plasma and CSF.^13-16^ Emerging evidence from study of other neurodegenerative diseases suggests that inflammation is not simply a passive response to neuronal death, but that it directly contributes to disease pathogenesis. Excessive inflammation can damage the brain in several ways, for example, through production of reactive oxygen and nitrogen species, which are toxic to neurons.^17,18^ Excessive microglial activation can also lead to aberrant phagocytosis of healthy neurons or synapses, which is linked to cognitive decline in mouse models of Alzheimer’s disease and aging.^19,20^ Despite the potential importance of neuroinflammation to disease pathogenesis, the causes of immune cell activation and inflammation in C9FTD/ALS are unknown.

One mechanism through which microglia and other immune cells can drive inflammation is via the NOD-LRR- and pyrin domain-containing 3 (NLRP3) inflammasome. The NLRP3 inflammasome is a multi-protein complex which forms in immune cells in response to stimuli such as pathogens, damage-associated molecular patterns (DAMPs) and particulates such as aggregating proteins. The NLRP3 inflammasome complex consists of three key components: NLRP3, which is the sensor molecule the inflammasome is named after, an adapter protein called ASC (apoptosis-associated speck-like protein containing a CARD), and pro-caspase-1, which mediates the downstream consequences of inflammasome activation. Assembly of the NLRP3 inflammasome complex triggers caspase-1-mediated cleavage of both pro-interleukin (IL)-1β and pro-IL-18 to produce the pro-inflammatory cytokines, IL-1β and IL-18, which are released from the cell. Inflammasome activation also leads to cell death via pyroptosis. IL-1β is often described as a ‘master’ pro-inflammatory cytokine, due to its wide range of functions in the innate immune system and ability to trigger a local inflammatory response.^21,22^ Excessive IL-1β release is known to be neurotoxic and is linked to a number of neurological diseases such as stroke and Alzheimer’s disease^23-26^

Recent work links the NLRP3 inflammasome to several neurodegenerative diseases including Alzheimer’s disease, Parkinson’s disease and ALS caused by SOD1 mutations^27-30^ Genetic or pharmacological inhibition of NLRP3 rescues cognitive impairments in different rodent models of Alzheimer’s disease,^26,31^ suggesting that the inflammasome can directly contribute to disease pathogenesis in dementia. Furthermore, a recent epidemiological study reported that long-term use of diclofenac, a non-steroidal anti-inflammatory drug which inhibits NLRP3 and is often prescribed for the treatment of conditions such as arthritis, is associated with reduced incidence of Alzheimer’s disease and slower cognitive decline.^32^ NLRP3 inhibiting drugs are therefore currently being investigated as a potential therapeutic strategy in Alzheimer’s disease. Here, we investigated the impact of DPRs arising from the *C9orf72* expansion on the NLRP3 inflammasome, to determine whether NLRP3 may contribute to the neuroinflammation observed in C9FTD/ALS patients.

## Materials and methods

### Mice

In-house colonies of wild-type (WT) C57BL/6 at the University of Manchester were maintained to provide primary cell cultures and *ex vivo* hippocampal slice cultures. Animals were allowed free access to food and water and maintained under light-, temperature- and humidity-controlled conditions. Both sexes of animals were used. All animal procedures adhered to the UK Animals (Scientific Procedures) Act (1986).

### Primary peritoneal macrophage preparation and treatment

Primary peritoneal macrophages were isolated from male and female adult WT mice. The peritoneal cavity was lavaged with RPMI 1640 media (Sigma) containing 5% v/v foetal bovine serum (FBS; Thermo), 25 mM 4-(2-hydroxyethyl)-1-piperazineethanesulfonic acid (HEPES) pH 7.3, 2 mM glutamine (Thermo), 100 U ml^−1^ penicillin (Thermo) and 100 μg ml^−1^ streptomycin (Thermo). Media were pooled from the lavages of four to five mice and centrifuged at 80×g for 10 min. Cell pellets were resuspended in Dulbecco’s modified Eagle media (DMEM; Sigma) containing 10% (v/v) FBS (Thermo), 100 U ml^−1^ penicillin (Thermo) and 100 μg ml^−1^ streptomycin (Thermo), plated at a density of 1 × 10^6^ cells ml^−1^ and incubated overnight at 37°C with 5% CO_2_. Macrophages were primed with 1 µg ml^−1^ lipopolysaccharide (LPS) (Sigma) for 4 h and media replaced with Opti-MEM (Sigma) prior to treatments. Cells were treated with 30 µM synthetic DPRs (GA15, GR15, PR15, AP15 or GP15 dissolved in sterile H_2_O; GenScript) and vehicle (H_2_O) and incubated for 24 h at 37°C with 5% CO_2_. Nigericin treatments (10 µM; Sigma) were performed 1 h prior to harvest.

### Primary bone marrow-derived macrophage preparation and treatment

Primary bone marrow-derived macrophages (BMDMs) were isolated from male and female adult WT mice. Bone marrow was extracted from femurs by centrifugation at 10 000×g for 10 s, and red blood cells were lysed with ACK lysing buffer (Lonza). Cells were passed through a 70-µm pore strainer (Corning) and centrifuged at 1500×g for 5 min. The cell pellet was resuspended in DMEM (Sigma) containing 10% (v/v) FBS (Thermo), 100 U ml^−1^ penicillin (Thermo), 100 μg ml^−1^ streptomycin (Thermo) and 30% L929 mouse fibroblast-conditioned medium for 7 days at 37°C with 5% CO_2_. BMDMs were seeded overnight at a density of 1 × 10^6^ cells ml^−1^ before treatment. Cells were primed with 1 µg ml^−1^ LPS (Sigma) for 4 h and media replaced with Opti-MEM (Sigma) prior to treatments. Cells were pre-treated with 10 µM MCC950 (Sigma), 100 µM AC-YVAD-CMK (VWR), 100 nM bafilomycin A1 (Tocris), 100 µM mefenamic acid (Sigma), 100 µM flufenamic acid (Sigma), 125 µM dimethyl fumarate (Sigma), 10 µM NS3728 (Sigma), 50 mM potassium gluconate (Sigma) or vehicle for 15 min, then treated with 30 µM synthetic GR_15_ (GenScript) or vehicle (H_2_O) and incubated for 24 h at 37°C. Nigericin treatments (10 µM; Sigma) were performed 1 h prior to harvest.

### Primary microglia preparation and treatment

Primary microglia were isolated from male and female adult WT mice. Mice were perfused with ice-cold Hank’s balanced salts solution (HBSS) and brains dissected out and stored in cold HBSS. The cerebellum and meningeal layers were removed before dicing the brain and centrifuging at 300×g for 2 min at 4°C. A MACS Neural Tissue Dissociation Kit (Miltenyi Biotec) was used according to manufacturer’s instructions to enzymatically process brain tissue, before homogenization with a Dounce homogenizer and centrifuging for 5 min at 400×g and 4°C. Cell pellets were resuspended in 30% v/v Percoll (supplier) in HBSS and centrifuged for 10 min at 700×g and 4°C with the brake set to the lowest setting to separate myelin, which was removed by aspiration. Microglia were isolated using magnetic CD11b + beads (Miltenyi Biotec) and seeded on plates coated with poly-L-lysine (supplier) at a density of 1.7 × 105 cells ml^−1^ in DMEM (Sigma) containing 10% (v/v) FBS (Thermo), 100 U ml^−1^ penicillin (Thermo) and 100 μg ml^−1^ streptomycin (Thermo), supplemented with 10 ng ml^−1^ of recombinant mouse M-CSF (R&D Systems, Abingdon, UK), and 50 ng ml^−1^ of recombinant human TGF-β. Cells were cultured for 7 days at 37°C with 5% CO_2_, with a media change after 3 days. After 7 days, cells were primed with 1 µg ml^−1^ LPS (Sigma) for 4 h and media replaced with Opti-MEM (Sigma) prior to treatments. Cells were treated with 30 µM synthetic DPRs (GA15, GR15, PR15, AP15 or GP15; GenScript), vehicle (H_2_O) or 5 mM adenosine triphosphate (ATP; Sigma) as a positive control and incubated for 24 h at 37°C.

### Hippocampal slice culture preparation and treatment

WT mouse pups of either sex aged 7 days were killed by cervical dislocation, and the brains were collected in phosphate-buffered saline (PBS) containing 5 mg ml^−1^ glucose. The hippocampi were dissected, and 400-μm slices were prepared using a McIlwain tissue chopper (Brinkman Instruments). Hippocampal slices were collected and placed on 0.4-μm Millicell culture inserts (Merck Millipore), as described previously.^33^ Slices were cultured in 1 ml MEM (Gibco) containing 20% (v/v) horse serum (Sigma), HEPES (30 mM; Fisher) and insulin (0.1 mg ml^−1^; pH 7.2–7.3; Gibco) and incubated at 37°C with 5% CO_2_. The culture media were changed every 2 days and slices were used at day 7. Hippocampal slices were primed with LPS (1 µg ml^−1^) for 3 h. The culture media were replaced with serum-free MEM with or without MCC950 (10 µM, 15 min). Vehicle (saline), GR (300 µM) or AP (300 µM) was then added to the culture media for 24 h, with media sampled at 4 h.

### Detection of cytokines

Culture media from cells and hippocampal slices were harvested on ice and IL-1β detected by ELISA and western blotting. For ELISA, the mouse IL-1β DuoSet kit (R&D Systems) was used according to manufacturer’s instructions. For western blotting, culture media were mixed with Laemmli buffer and proteins separated by electrophoresis on 4–15% polyacrylamide gels (BioRad) and then transferred to nitrocellulose membrane (Whatman) at 15 V for 1 h. Membranes were blocked with 5% w/v bovine serum albumin (BSA; Roche) in Tris-buffered saline with 0.1% v/v Tween-20 (TBS-T) for 1 h at room temperature before incubation overnight at 4°C in 250 ng ml^−1^ goat anti-mouse IL-1β primary antibody (R&D Systems, AF-401-NA) or 1.7 µg ml^−1^ rabbit anti-mouse caspase-1 primary antibody (Abcam, ab179515) in 5% w/v BSA in TBS-T. Membranes were washed and incubated with rabbit anti-goat IgG secondary antibody (500 ng ml^−1^ in 5% w/v milk in TBS-T; Agilent, P044901-2) for 1 h at room temperature. Proteins were visualized using Amersham ECL Western Blotting Detection Reagent (GE Healthcare) and a G:Box imager and GeneSys software (SynGene).

### Cytotoxicity assays

Lactate dehydrogenase levels in culture media were quantified as a measure of cell death using the CytoTox 96 Non-Radioactive Cytotoxicity Assay kit (Promega) according to manufacturer’s instructions.

### Immunofluorescence imaging of hippocampal slices

Hippocampal slices were washed once with cold PBS and fixed in 4% PFA for 1 h at 4°C. Slices were then washed twice in cold PBS and then incubated with rabbit anti-mouse ASC (202 ng ml^−1^; CST) primary antibody overnight at 4°C. Hippocampal slices were washed and incubated with Alexa Fluor^™^ 488 donkey anti-rabbit IgG (2 µg ml^−1^; Invitrogen) secondary antibody for 2 h at room temperature. All antibody incubations were performed using PBS 0.3% Triton X-100. Wash steps were performed using PBS 0.1% Tween unless stated otherwise. Slices were washed and then incubated in 4′,6-diamidino-2-phenylindole (DAPI) (0.5 µg ml^−1^, 15 min; Sigma) at room temperature before final washing in distilled H_2_O and mounting using ProLong^™^ gold antifade mountant (Thermo) prior to imaging using widefield microscopy. Images were collected on a Zeiss Axioimager.M2 upright microscope using a 5× or 20× Plan Apochromat objective and captured using a Coolsnap HQ2 camera (Photometrics) through Micromanager software (v1.4.23). Specific band-pass filter sets for DAPI and FITC were used to prevent bleed-through from one channel to the next. Analysis was performed using FIJI (ImageJ) on images acquired from three regions of up to three separate hippocampal slices (from the same insert) per treatment, and these values were averaged for each biological repeat. ASC speck formation was quantified on 20× widefield microscopy images by subtracting background, manually setting thresholds and analysing particles with the following parameters: size 1–10 μm^2^ and circularity 0.9–1.0.

### Statistical analysis

All data were analysed using GraphPad Prism version 9.1.2 using appropriate statistical tests such as one-way ANOVA with *post hoc* tests for multiple comparisons such as Tukey’s and Dunnett’s tests. Equal variance and normality were assessed with the Levene’s test and the Shapiro–Wilk test, respectively, and appropriate transformations were applied when necessary. Primary cell preparations from different mice or hippocampal slices cultured from different litters were considered independent replicates, with all experiments performed in triplicate as minimum (*n* numbers for each experiment detailed in figure legends).

## Results

**Figure 1:**
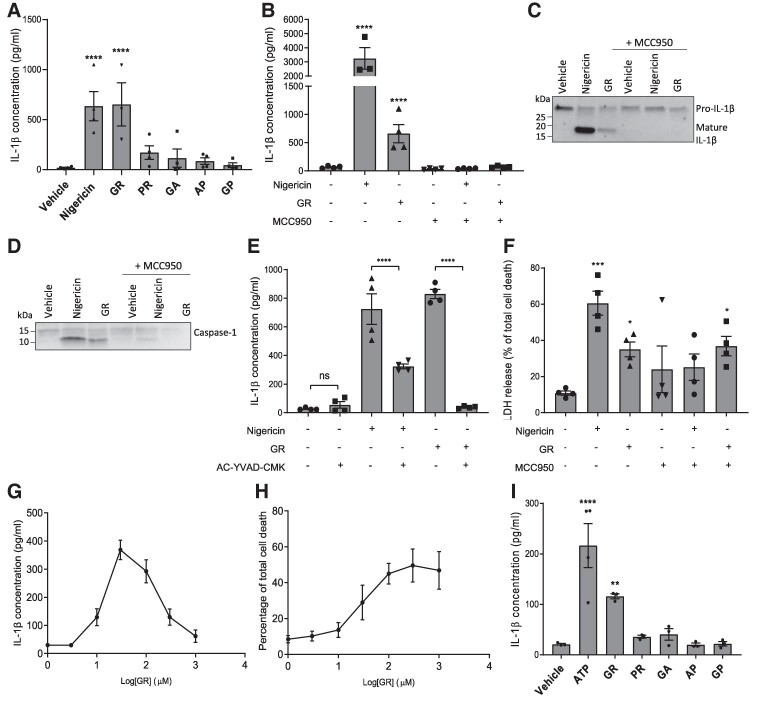
**GR activates the NLRP3 inflammasome in macrophages and microglia.** (**A)** LPS-primed primary peritoneal macrophages from male and female WT mice were treated with 30 μM DPRs or controls for 24 h (*n* = 4). IL-1β was measured in the media by ELISA. (**B**) GR increased IL-1β release in LPS-primed BMDMs (*n* = 4) and was prevented by MCC950. (**C**) Western blot of culture media from GR-treated bone marrow-derived macrophages (BMDMs) showing pro-IL-1β cleavage to produce an active fragment (∼17 kDa). Nigericin was used as a positive control (*n* = 3). (**D**) Western blot of culture media from GR-treated bone marrow-derived macrophages (BMDMs) showing pro-caspase-1 cleavage to produce an active fragment (∼10 kDa). Nigericin was used as a positive control (*n* = 3). (**E**) GR-induced IL-1β secretion from LPS-primed BMDMs was prevented by AC-YVAD-CMK pre-treatment (*n* = 4). (**F**) Quantification of cell death in GR-treated BMDMs by LDH assay, 24 h post-treatment. (**G**) Dose–response curve of IL-1β detected in BMDM culture media 24 h after treatment with increasing concentrations of GR. (**H**) Dose–response curve of BMDM cell death measured by LDH assay, 24 h after treatment with increasing concentrations of GR. (**I**) IL-1β levels in culture media of LPS-primed primary WT mouse microglia 24 h post-treatment with 30 μM DPRs or 5 mM ATP (*n* = 3). All data analysed by one-way ANOVA with *post hoc* Dunnett’s or Tukey’s tests for multiple comparisons. **** indicates *P* < 0.0001, *** indicates *P* < 0.001, and ** indicates *P* 0.01. All values are mean ± SEM. Individual mice were considered the experimental unit. See Supplementary Fig. 1 for uncropped blots.

To determine whether DPRs arising from the *C9orf72* expansion activate the NLRP3 inflammasome, LPS-primed primary peritoneal macrophages isolated from WT mice were treated with synthetic DPRs. Nigericin, a known NLRP3 activator, was used as a positive control. Release of IL-1β into the culture media was quantified by ELISA after a 24-h treatment (Fig. 1A). Four of the five DPRs, GA, PR, AP and GP, did not affect IL-1β secretion. However, the final DPR, GR, caused a significant increase in IL-1β secretion compared to vehicle-treated control. To confirm that this was not a cell-specific artefact, we next treated a different type of macrophage cell, primary mouse BMDMs, with GR and quantified IL-1β release after 24 h. GR caused significant IL-1β release, which was inhibited by pre-treatment with MCC950, a well-characterized NLRP3 selective inhibitor,^34^ confirming the response was mediated via the NLRP3 inflammasome (Fig. 1B). Western blotting of the culture media confirmed the presence of both mature IL-1β with a band at ∼17 kDa and mature caspase-1 with a band at ∼10 kDa following GR treatment, which were not present if cells were pre-treated with MCC950 (Fig. 1C-D; uncropped blot images in Supplemental Fig. 1A and B). Furthermore, GR-induced IL-1β release was prevented by pre-treatment with AC-YVAD-CMK, a caspase-1 selective inhibitor (Fig. 1E). Cell death was also quantified as lactate dehydrogenase (LDH) release (Fig. 1F). GR significantly increased LDH release from BMDMs and was not inhibited by MCC950, suggesting that GR is toxic to macrophages independently of NLRP3 activation.

Next, we investigated whether there was a dose–response relationship between GR concentration and NLRP3 inflammasome activation. Primary BMDMs were treated with increasing doses of GR peptide for 24 h, and IL-1β secretion was quantified by ELISA. Cell death was also measured by LDH assay. A clear dose–response relationship was observed for GR-induced IL-1β release for the lower concentrations of GR tested; however, the response peaked at 30 µM GR treatment, after which IL-1β release began to decrease with increasing GR concentration (Fig. 1G). Interestingly, the severity of cell death continued to increase with increasing GR concentration even beyond the point at which IL-1β secretion began to decline (Fig. 1H). This confirms our findings in Fig. 1F that GR is toxic to macrophages independently of NLRP3, and suggests that at higher concentrations GR may kill cells too quickly for NLRP3 activation to occur, explaining the decline in IL-1β release above 30 µM GR treatment. Nevertheless, GR triggered IL-1β from macrophages in a dose-dependent manner at lower treatment concentrations, before this toxicity threshold was reached.

Taken together, these data demonstrate that GR activates the NLRP3 inflammasome. In order to establish the relevance of our findings to FTD/ALS, we next investigated whether DPRs also activate NLRP3 in microglia, the main immune cells found in the central nervous system. Primary microglia were isolated from adult WT mice and treated with GR, GA, PR, AP or GP for 24 h, and IL-1β secretion quantified by ELISA (Fig. 1I). As observed in macrophages, GR, but not the other DPRs, significantly increased IL-1β release from microglia. This highlights the relevance of our findings to the brain and suggests that *C9orf72* DPRs could directly contribute to the neuroinflammation observed in FTD/ALS patients.

**Figure 2:**
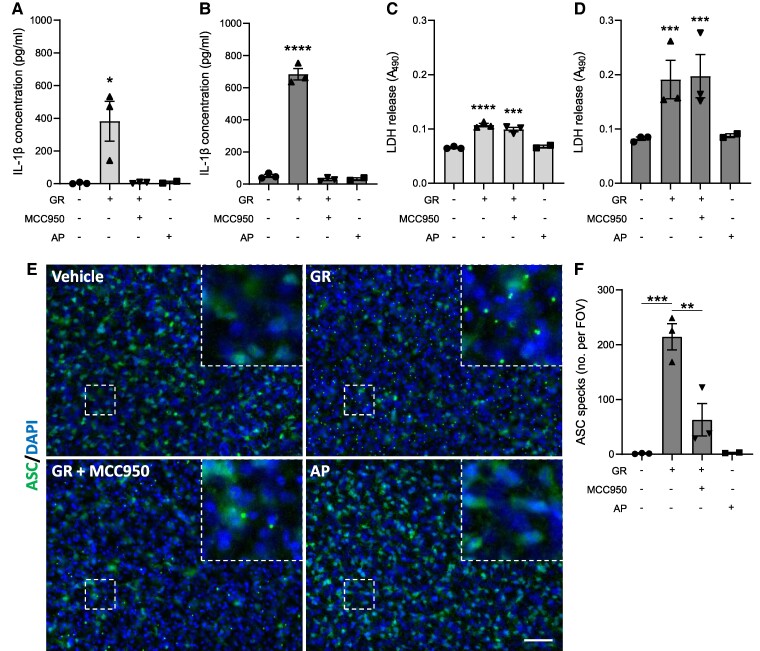
**GR activates the NLRP3 inflammasome in hippocampal slice cultures.** Mouse hippocampal slice cultures (from male and female mice) were LPS-primed and treated with GR ± MCC950 for 24 h (*n* = 3). AP treatment was also performed (*n* = 2). IL-1β concentrations in media collected from hippocampal slices 4 h (**A**) and 24 h (**B**) post-treatment. Cell death measured by LDH assay of media collected from hippocampal slices 4 h (**C**) and 24 h (**D**) post-treatment. (**E**) Immunofluorescence imaging of ASC in hippocampal slices. ASC specks are visible as intense puncta in the cytosol of cells in GR-treated hippocampal slices (top right). (**F**) Quantification of total ASC specks per field of vision. All values are mean ± SEM. Data were analysed by one-way ANOVA with *post hoc* Dunnett’s or Tukey’s tests for multiple comparisons. The scale bars represent 50 µm. Individual mice were considered the experimental unit.

Since the data described above were obtained using monocultures of specific cell types, we next investigated whether GR would activate the inflammasome in a more complex system containing multiple brain cell types. To achieve this, we used *ex vivo* hippocampal slice cultures from WT mice, which retain some 3D neuronal architecture and contain microglia, astrocytes and other brain cell types.^35^ Cultured brain slices were treated with GR or vehicle for 4 or 24 h and IL-1β secretion measured by ELISA of the culture media. A different DPR, AP, was used as an additional control to confirm that any changes observed were not generic effects of any foreign peptide applied to the slices. GR significantly increased IL-1β concentration in the culture media after 4 h (Fig. 2A), with a further increase after 24 h (Fig. 2B). Pre-treatment with MCC950 prevented IL-1β secretion at both timepoints, confirming that this release was NLRP3-dependent. GR also caused cause cell death in hippocampal slices at both 4 h (Fig. 2C) and 24 h (Fig. 2D) post-treatment. As with BMDM cultures, cell death was not reduced by MCC950 treatment. AP treatment did not impact IL-1β secretion at either timepoint, confirming the response was GR-specific. Furthermore, AP did not cause significant cell death compared to vehicle.

In addition to quantifying IL-1β release, immunofluorescence imaging was also performed on GR-treated hippocampal slices using an antibody against ASC (Fig. 2E). The number of small, punctate points of intense ASC staining (ASC specks) observed per field of vision was significantly increased in GR-treated slice tissue compared to control (quantification in Fig. 2F), indicating the formation of ASC specks in response to GR. Specks were not formed in AP-treated slices. Treatment with MCC950 reduced the number of specks induced by GR, confirming that the speck formation was NLRP3-dependent.

**Figure 3:**
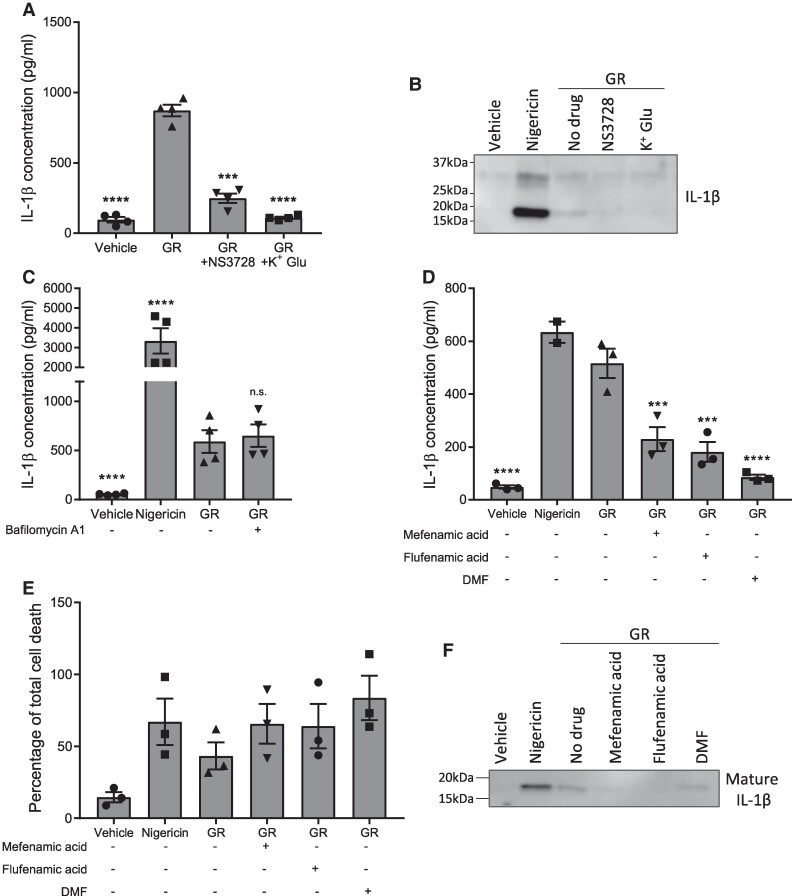
**Characterization of GR-induced NLRP3 inflammasome activation.** (**A**), (**B**) LPS-primed primary mouse BMDMs (from male and female mice) were pre-treated for 15 min with a chloride channel inhibitor, NS3728, or potassium gluconate (K^+^ Glu) prior to a 24-h treatment with GR. IL-1β concentrations in the culture media were measured by ELISA (*n* = 4) (**A**) and cleavage of pro-IL-1β assessed by WB of the media (*n* = 3) (**B**). **C** LPS-primed BMDMs were pre-treated for 15 min with bafilomycin A1 or vehicle prior to a 24-h treatment with GR, and IL-1β secretion quantified by ELISA (*n* = 4). (**D**)**–**(**F**) LPS-primed mouse primary BMDMs were pre-treated for 15 min with one of three clinically approved drugs which are known NLRP3 inhibitors: mefenamic acid, flufenamic acid, and dimethyl fumarate (DMF), before a 24-h treatment with GR. (**D**) Mean IL-1β concentrations in the culture media as detected by ELISA (*n* = 3). (**E**) Quantification of cell death in BMDMs assessed by LDH assay (*n* = 3). (**F**) Western blotting of the culture media showing cleavage of pro-IL-1β to produce mature IL-1β (17 kDa) (*n* = 3). All data were analysed by one-way ANOVA with *post hoc* Dunnett’s or Tukey’s tests for multiple comparisons. The error bars indicate SEM. Individual mice were considered the experimental unit. See Supplementary Fig. 2 for uncropped blots.

The NLRP3 inflammasome is activated by multiple pathways. Canonical, non-canonical and alternative pathways of inflammasome activation have all been described.^36,37^ As such, we next sought to further characterize GR-induced inflammasome activation. Firstly, we aimed to determine whether the process is dependent on K^+^ or Cl^−^ movement across the cell membrane, as has been demonstrated in cases of canonical NLRP3 activation.^38,39^ Prior to GR treatment, BMDMs were pre-treated with either a Cl^−^ channel inhibitor, NS3728, or high concentration potassium gluconate to prevent K^+^ efflux from the cell. Both NS3728 and potassium gluconate treatment reduced IL-1β secretion in response to GR (Fig. 3A). Western blotting confirmed a reduction in IL-1β cleavage in media from cells pre-treated with either NS3728 or potassium gluconate compared to GR alone (Fig. 3B; uncropped blot image in Supplemental Fig. 2A). Therefore, GR-induced NLRP3 activation is sensitive to both K^+^ and Cl^−^ movement. We also pre-treated BMDMs with bafilomycin A1, a vacuolar H^+^ ATPase inhibitor which prevents autophagy.^40^ Bafilomycin A1 is known to modulate the alternative pathway of NLRP3 inflammasome activation in monocytes.^41^ However, no difference was observed in IL-1β release from BMDMs in response to GR with or without bafilomycin A1 pre-treatment (Fig. 3C), suggesting that GR activated NLRP3 via the canonical route.

Finally, we investigated whether several existing drugs which are already licenced for clinical use, and which are known to inhibit NLRP3,^31,42^ could prevent GR-induced NLRP3 inflammasome activation. LPS-primed BMDMs were pre-treated with either mefenamic acid, flufenamic acid or dimethyl fumarate prior to a 24-h treatment with GR. Mefenamic acid and flufenamic acid are non-steroidal anti-inflammatory drugs currently used to treat pain and inflammation in conditions such as rheumatoid arthritis and osteoarthritis.^43^ Dimethyl fumarate is a drug used to treat multiple sclerosis.^44,45^ All three drugs have previously been shown to also inhibit the NLRP3 inflammasome.^31,42^ None of the drugs prevented GR toxicity; however, all three treatments inhibited GR-induced inflammasome activation, as measured by IL-1β ELISA and western blotting of the culture media (Fig. 3D-F; uncropped blot image in Supplemental Fig. 2B). Therefore, there are several drugs already clinically approved for other conditions which could potentially be repurposed to target NLRP3-dependent inflammation in FTD/ALS.

## Discussion

The data presented here suggest that GR, one of the five DPRs arising from unconventional translation of the *C9orf72* expansion, is an NLRP3 inflammasome activator. GR-induced NLRP3 activation caused release of the pro-inflammatory cytokine, IL-1β, from macrophages and microglia. This could at least partially explain the neuroinflammation observed in patients with the *C9orf72* expansion and highlights NLRP3 as a potential therapeutic target for C9FTD/ALS. We also demonstrate that GR-induced NLRP3 inflammasome activation is sensitive to both potassium and chloride current inhibitors and leads to formation of ASC specks, suggesting a canonical mechanism of activation.

In addition, we found that GR was toxic to both microglia and macrophages and that this was not dependent on NLRP3 inflammasome activation. This is unsurprising, since GR has previously been found to be toxic in multiple other cells types, and is known to cause multiple phenotypes such as impaired nucleocytoplasmic transport, ribosomal inhibition, nucleolar stress and altered stress granule dynamics^5,7-9,46^ However, the mechanisms through which GR kills microglia and macrophages remain unknown. Since GR toxicity was not NLRP3-dependent, there are two possible explanations: GR may disrupt microglial cellular function in such a way that triggers inflammasome activation shortly before killing the cell, or GR may be toxic to microglia in a way that does not directly activate the inflammasome, but that the DAMPs released following cell death go on to trigger inflammasome activation in neighbouring microglia. Further investigation to fully understand the mechanisms through which GR activates the NLRP3 inflammasome will be useful and may aid future drug discovery for FTD/ALS.

Regardless of the mechanisms underlying GR-induced inflammasome activation, the data presented here support a role of NLRP3 in C9FTD/ALS. Our findings are corroborated by recent clinical studies which found elevated levels of both IL-1β^14^ and IL-18^47^ in the CSF of patients with C9ALS compared to healthy controls. A meta-analysis of 25 studies including 812 ALS patients and 639 control subjects found that IL-1β levels are also increased in blood plasma in ALS.^13^ Interestingly, a negative correlation has also been observed between plasma IL-1β levels and survival time in C9ALS patients, suggesting that IL-1β may exacerbate disease.^48^ In addition, mice expressing GR exhibit microgliosis, indicating that GR is sufficient to cause inflammation *in vivo* in the absence of other pathological features of C9FTD/ALS.^8^ Of note, microgliosis occurred early in the disease process in GR mice, prior to the development of cognitive and motor deficits. This implies that neuroinflammation is an early feature of FTD/ALS, and therefore could contribute to disease pathogenesis.

A recent study reports that a different *C9orf72* DPR, PR, can activate the NLRP3 inflammasome in HMC3 cells, an immortalized microglial line, resulting in IL-1β and IL-18 release.^49^ This study did not report findings on GR or other DPRs. Conditioned media from PR-expressing microglia are also toxic when applied to cultured motor neurons, implicating the PR-induced immune response in neurodegeneration.^49^ This is in contrast to our findings that PR did not activate the inflammasome in either microglia or macrophages. These conflicting findings may be due to the longer repeat-length of PR (50 repeats) used by Fu *et al*. We have previously demonstrated that DPR repeat-length determines the toxicity in other cell types, with longer repeat DPRs causing more severe phenotypes.^5,6^ We also found GR to be the most toxic DPR in other models, with a shorter repeat-length required to cause various phenotypes. Therefore, it is possible that our negative data were due to the relatively short repeat-length of PR used and that PR can activate the inflammasome if expressed at longer lengths. Longer repeat-length mouse models expressing PR using adeno-associated virus (AAV) injections now exist and would be useful for future studies to corroborate that PR consistently activates the inflammasome at these lengths. A recent study using AAVs to express GA in mice reports increased NLRP3 and pro-IL-1β expression and caspase-1 cleavage in brain, suggesting that GA may also activate the NLRP3 inflammasome.^50^ As with PR, the conflict with our finding that GA did not activate the inflammasome may be explained by differing repeat-lengths. Deletion of the NLRP3 gene prevented motor impairments caused by GA expression in mice, suggesting that the inflammasome may actively contribute to neuronal dysfunction or degeneration in FTD/ALS.^50^

NLRP3 is linked to other forms of FTD/ALS not caused by the *C9orf72* expansion. This is most studied in models of ALS caused by *SOD1* mutations. Treatment with mutant SOD1 peptide causes IL-1β secretion from WT but not NLRP3−/− primary mouse microglia.^51^ In addition, *SOD1* mutant mice exhibit elevated mRNA and protein expression levels of NLRP3, ASC and IL-1β in both brain^52,53^ and skeletal muscle,^30^ along with microgliosis early in the disease process. Caspase-1 expression is also elevated in the spinal cord of SOD1 mutant mice.^54^ Similarly, protein expression of NLRP3, activated caspase-1, IL-18 and IL-1β are all increased in mutant SOD1 rats.^55^ Interestingly, NLRP3 activation is also found to occur in astrocytes in SOD1 mice.^53^ Elevated expression of NLRP3 markers in astrocytes is also observed in brain tissue from sporadic ALS patients, and immunohistochemical analysis shows co-localization between NLRP3 and the astrocytic marker, GFAP.^53^

Several recent studies suggest the NLRP3 inflammasome is also activated by TDP-43, an RNA-binding protein which forms pathological intraneuronal inclusions in the majority of cases of FTD/ALS.^56^ Upregulation of NLRP3, ASC and caspase-1 mRNA is observed in the spinal cord of mice expressing an ALS-linked TDP-43 mutation, and treatment with WT or mutant TDP-43 peptide causes IL-1β release in mouse primary microglia. This was rescued by pre-treatment with MCC950.^51^ Furthermore, TDP-43 treatment is found to only be toxic to motor neurons in the presence of microglia, suggesting that TDP-43-induced NLRP3 activation may contribute to neurodegeneration in FTD/ALS.^57^ Finally, NLRP3 is also linked to FTD caused by tau mutations; phosphorylated tau peptide activates the NLRP3 inflammasome in cultured microglia^58,59^ and in mouse models expressing mutant tau.^60^ Importantly, these findings are validated by western blotting analysis of FTD-tau patient brain, which found elevated levels of ASC, IL-1β and cleaved caspase-1 in the cortex.^60^

## Conclusion

Taken together, our findings combined with the published literature suggest that NLRP3 inflammasome activation is a common feature of multiple subtypes of FTD and ALS, with different aggregating proteins triggering the response in each case. This offers NLRP3 inhibition as a promising therapeutic strategy for multiple types of FTD/ALS. Several drugs which inhibit NLRP3 already exist and are clinically approved for use in other conditions.^31,42^ However, a gap in our knowledge remains regarding the contribution of NLRP3 to disease pathogenesis. Genetic or pharmacological inhibition of NLRP3 has recently been shown to rescue cognitive impairment in mouse models of FTD-tau, supporting the possibility of NLRP3 inhibition as a useful therapeutic strategy.^60,61^ NLRP3 is also linked to tau seeding and aggregation, with NLRP3 inhibition reducing pathological burden in tau transgenic mice.^59,60^ Further work is required to determine the contribution of GR-induced NLRP3 activation to disease pathogenesis in C9FTD/ALS.

## Supplementary Material

fcae282_Supplementary_Data

## Data Availability

All data supporting the findings of this study are contained within the article.

## References

[1] Renton AE, Majounie E, Waite A, et al A hexanucleotide repeat expansion in *C9ORF72* is the cause of chromosome 9p21-linked ALS-FTD. Neuron. 2011;72(2):257–268. 21944779 10.1016/j.neuron.2011.09.010 PMC3200438

[2] DeJesus-Hernandez M, Mackenzie IR, Boeve BF, et al Expanded GGGGCC hexanucleotide repeat in noncoding region of *C9ORF72* causes chromosome 9p-linked FTD and ALS. Neuron. 2011;72(2):245–256. 21944778 10.1016/j.neuron.2011.09.011 PMC3202986

[3] Mori K, Weng SM, Arzberger T, et al The *C9orf72* GGGGCC repeat is translated into aggregating dipeptide-repeat proteins in FTLD/ALS. Science. 2013;339(6125):1335–1338. 23393093 10.1126/science.1232927

[4] Ash PEA, Bieniek KF, Gendron TF, et al Unconventional translation of *C9ORF72* GGGGCC expansion generates insoluble polypeptides specific to c9FTD/ALS. Neuron. 2013;77(4):639–646. 23415312 10.1016/j.neuron.2013.02.004 PMC3593233

[5] Callister B, Ryan J, Sim S, Rollinson J, Pickering-Brown S, M S. Modelling *C9orf72* dipeptide repeat proteins of a physiologically relevant size. Hum Mol Genet. 2016;25(23):5069. 27798094 10.1093/hmg/ddw327 PMC5886041

[6] Swaminathan A, Bouffard M, Liao M, et al Expression of *C9orf72*-related dipeptides impairs motor function in a vertebrate model. Hum Mol Genet. 2018;27(10):1754–1762. 29528390 10.1093/hmg/ddy083 PMC5932562

[7] Mizielinska S, Grönke S, Niccoli T, et al *C9orf72* repeat expansions cause neurodegeneration in Drosophila through arginine-rich proteins. Science. 2014;345(6201):1192–1194. 25103406 10.1126/science.1256800 PMC4944841

[8] Zhang YJ, Gendron TF, Ebbert MTW, et al Poly(GR) impairs protein translation and stress granule dynamics in *C9orf72*-associated frontotemporal dementia and amyotrophic lateral sclerosis. Nat Med. 2018;24(8):1136–1142. 29942091 10.1038/s41591-018-0071-1 PMC6520050

[9] Ryan S, Rollinson S, Hobbs E, Pickering-Brown S. *C9orf72* dipeptides disrupt the nucleocytoplasmic transport machinery and cause TDP-43 mislocalisation to the cytoplasm. Sci Rep. 2022;12(1):4799. 35314728 10.1038/s41598-022-08724-w PMC8938440

[10] West RJH, Sharpe JL, Voelzmann A, et al Co-expression of *C9orf72* related dipeptide-repeats over 1000 repeat units reveals age- and combination-specific phenotypic profiles in Drosophila. Acta Neuropathol Commun. 2020;8(1):158. 32894207 10.1186/s40478-020-01028-y PMC7487709

[11] Lant SB, Robinson AC, Thompson JC, et al Patterns of microglial cell activation in frontotemporal lobar degeneration. Neuropathol Appl Neurobiol. 2014;40(6):686–696. 24117616 10.1111/nan.12092

[12] Cagnin A, Rossor M, Sampson EL, MacKinnon T, Banati RB. In vivo detection of microglial activation in frontotemporal dementia. Ann Neurol. 2004;56(6):894–897. 15562429 10.1002/ana.20332

[13] Hu Y, Cao C, Qin XY, et al Increased peripheral blood inflammatory cytokine levels in amyotrophic lateral sclerosis: A meta-analysis study. Sci Rep. 2017;7(1):9094. 28831083 10.1038/s41598-017-09097-1 PMC5567306

[14] Pinilla G, Kumar A, Floaters MK, Pardo CA, Rothstein J, Ilieva H. Increased synthesis of pro-inflammatory cytokines in *C9ORF72* patients. Amyotroph Lateral Scler Front Degener. 2021;22(7–8):517–527. 10.1080/21678421.2021.1912100 33929933

[15] Katisko K, Solje E, Korhonen P, et al Peripheral inflammatory markers and clinical correlations in patients with frontotemporal lobar degeneration with and without the *C9orf72* repeat expansion. J Neurol. 2020;267(1):76–86. 10.1007/s00415-019-09552-1 PMC6954907 31559531

[16] Italiani P, Carlesi C, Giungato P, et al Evaluating the levels of interleukin-1 family cytokines in sporadic amyotrophic lateral sclerosis. J Neuroinflammation. 2014;11:94. 24884937 10.1186/1742-2094-11-94 PMC4039322

[17] Emerit J, Edeas M, Bricaire F. Neurodegenerative diseases and oxidative stress. Biomed Pharmacother. 2004;58(1):39–46. 14739060 10.1016/j.biopha.2003.11.004

[18] Block ML, Zecca L, Hong JS. Microglia-mediated neurotoxicity: Uncovering the molecular mechanisms. Nat Rev Neurosci. 2007;8(1):57–69. 17180163 10.1038/nrn2038

[19] Hong S, Beja-Glasser VF, Nfonoyim BM, et al Complement and microglia mediate early synapse loss in Alzheimer mouse models. Science. 2016;352(6286):712–716. 27033548 10.1126/science.aad8373 PMC5094372

[20] Shi Q, Colodner KJ, Matousek SB, et al Complement C3-deficient mice fail to display age-related hippocampal decline. J Neurosci. 2015;35(38):13029–13042. 26400934 10.1523/JNEUROSCI.1698-15.2015 PMC6605437

[21] Katsnelson MA, Lozada-Soto KM, Russo HM, Miller BA, Dubyak GR. NLRP3 inflammasome signaling is activated by low-level lysosome disruption but inhibited by extensive lysosome disruption: Roles for K+ efflux and Ca2 + influx. Am J Physiol Cell Physiol. 2016;311:C83–C100. 27170638 10.1152/ajpcell.00298.2015 PMC4967136

[22] Dinarello CA . Immunological and inflammatory functions of the interleukin-1 family. Annu Rev Immunol. 2009;27:519–550. 19302047 10.1146/annurev.immunol.021908.132612

[23] Allan SM, Tyrrell PJ, Rothwell NJ. Interleukin-1 and neuronal injury. Nat Rev Immunol. 2005;5(8):629–640. 16034365 10.1038/nri1664

[24] Greenhalgh AD, Brough D, Robinson EM, Girard S, Rothwell NJ, Allan SM. Interleukin-1 receptor antagonist is beneficial after subarachnoid haemorrhage in rat by blocking haem-driven inflammatory pathology. Dis Model Mech. 2012;5(6):823–833. 22679224 10.1242/dmm.008557 PMC3484865

[25] Thornton P, Pinteaux E, Gibson RM, Allan SM, Rothwell NJ. Interleukin-1-induced neurotoxicity is mediated by glia and requires caspase activation and free radical release. J Neurochem. 2006;98(1):258–266. 16805812 10.1111/j.1471-4159.2006.03872.x

[26] Heneka MT, Kummer MP, Stutz A, et al NLRP3 is activated in Alzheimer’s disease and contributes to pathology in APP/PS1 mice. Nature. 2013;493(7434):674–678. 23254930 10.1038/nature11729 PMC3812809

[27] Codolo G, Plotegher N, Pozzobon T, et al Triggering of inflammasome by aggregated α–synuclein, an inflammatory response in synucleinopathies. PLoS One. 2013;8(1):e55375. 23383169 10.1371/journal.pone.0055375 PMC3561263

[28] Gordon R, Albornoz EA, Christie DC, et al Inflammasome inhibition prevents α-synuclein pathology and dopaminergic neurodegeneration in mice. Sci Transl Med. 2018;10(465):eaah4066. 30381407 10.1126/scitranslmed.aah4066 PMC6483075

[29] White CS, Lawrence CB, Brough D, Rivers-Auty J. Inflammasomes as therapeutic targets for Alzheimer’s disease. Brain Pathol. 2017;27(2):223–234. 28009077 10.1111/bpa.12478 PMC8029266

[30] Moreno-García L, Miana-Mena FJ, Moreno-Martínez L, et al Inflammasome in ALS skeletal muscle: NLRP3 as a potential biomarker. Int J Mol Sci. 2021;22(5):1–15. 10.3390/ijms22052523 PMC7959138 33802349

[31] Daniels MJD, Rivers-Auty J, Schilling T, et al Fenamate NSAIDs inhibit the NLRP3 inflammasome and protect against Alzheimer’s disease in rodent models. Nat Commun. 2016;7:12504. 27509875 10.1038/ncomms12504 PMC4987536

[32] Rivers-Auty J, Mather AE, Peters R, Lawrence CB, Brough D. Anti-inflammatories in Alzheimer’s disease-potential therapy or spurious correlate? Brain Commun. 2020;2(2):fcaa109. 33134914 10.1093/braincomms/fcaa109 PMC7585697

[33] Stoppini L, Buchs PA, Muller D. A simple method for organotypic cultures of nervous tissue. J Neurosci Methods. 1991;37(2):173–182. 1715499 10.1016/0165-0270(91)90128-m

[34] Coll RC, Robertson AAB, Chae JJ, et al A small-molecule inhibitor of the NLRP3 inflammasome for the treatment of inflammatory diseases. Nat Med. 2015;21(3):248–255. 25686105 10.1038/nm.3806 PMC4392179

[35] Hoyle C, Redondo-Castro E, Cook J, et al Hallmarks of NLRP3 inflammasome activation are observed in organotypic hippocampal slice culture. Nat Rev Immunol. 2020;161(1):39–52. 10.1111/imm.13221 PMC7450173 32445196

[36] Kayagaki N, Warming S, Lamkanfi M, et al Non-canonical inflammasome activation targets caspase-11. Nature. 2011;479(7371):117–121. 22002608 10.1038/nature10558

[37] Kelley N, Jeltema D, Duan Y, He Y. The NLRP3 inflammasome: An overview of mechanisms of activation and regulation. Int J Mol Sci. 2019;20(13):3328. 31284572 10.3390/ijms20133328 PMC6651423

[38] Muñoz-Planillo R, Kuffa P, Martínez-Colón G, Smith BL, Rajendiran TM, Núñez G. K+ efflux is the common trigger of NLRP3 inflammasome activation by bacterial toxins and particulate matter. Immunity. 2013;38(6):1142–1153. 23809161 10.1016/j.immuni.2013.05.016 PMC3730833

[39] Green JP, Yu S, Martín-Sánchez F, et al Chloride regulates dynamic NLRP3-dependent ASC oligomerization and inflammasome priming. Proc Natl Acad Sci U S A. 2018;115(40):E9371–E9380. 30232264 10.1073/pnas.1812744115 PMC6176575

[40] Mauvezin C, Neufeld TP. Bafilomycin A1 disrupts autophagic flux by inhibiting both V-ATPase-dependent acidification and Ca-P60A/SERCA-dependent autophagosome-lysosome fusion. Autophagy. 2015;11(8):1437. 26156798 10.1080/15548627.2015.1066957 PMC4590655

[41] Yu S, Green J, Wellens R, Lopez-Castejon G, Brough D. Bafilomycin A1 enhances NLRP3 inflammasome activation in human monocytes independent of lysosomal acidification. FEBS J. 2021;288(10):3186–3196. 33145969 10.1111/febs.15619 PMC8247003

[42] Hoyle C, Green JP, Allan SM, Brough D, Lemarchand E. Itaconate and fumarate derivatives inhibit priming and activation of the canonical NLRP3 inflammasome in macrophages. Nat Rev Immunol. 2022;165(4):460–480. 10.1111/imm.13454 PMC9426622 35137954

[43] Green GA . Understanding NSAIDs: From aspirin to COX-2. Clin Cornerstone. 2001;3(5):50–59. 11464731 10.1016/s1098-3597(01)90069-9

[44] Miglio G, Veglia E, Fantozzi R. Fumaric acid esters prevent the NLRP3 inflammasome-mediated and ATP-triggered pyroptosis of differentiated THP-1 cells. Int Immunopharmacol. 2015;28(1):215–219. 26096886 10.1016/j.intimp.2015.06.011

[45] Liu X, Zhou W, Zhang X, et al Dimethyl fumarate ameliorates dextran sulfate sodium-induced murine experimental colitis by activating Nrf2 and suppressing NLRP3 inflammasome activation. Biochem Pharmacol. 2016;112:37–49. 27184504 10.1016/j.bcp.2016.05.002

[46] Loveland AB, Svidritskiy E, Susorov D, et al Ribosome inhibition by *C9ORF72*-ALS/FTD-associated poly-PR and poly-GR proteins revealed by cryo-EM. Nat Commun. 2022;13(1):2776. 35589706 10.1038/s41467-022-30418-0 PMC9120013

[47] Huang F, Zhu Y, Hsiao-Nakamoto J, et al Longitudinal biomarkers in amyotrophic lateral sclerosis. Ann Clin Transl Neurol. 2020;7(7):1103. 32515902 10.1002/acn3.51078 PMC7359115

[48] Olesen MN, Wuolikainen A, Nilsson AC, et al Inflammatory profiles relate to survival in subtypes of amyotrophic lateral sclerosis. Neurol Neuroimmunol Neuroinflammation. 2020;7(3):e697. 10.1212/NXI.0000000000000697 PMC7136052 32123048

[49] Fu RH, Tsai CW, Chiu SC, et al C9-ALS-associated proline-arginine dipeptide repeat protein induces activation of NLRP3 inflammasome of HMC3 microglia cells by binding of complement component 1 Q subcomponent-binding protein (C1QBP), and syringin prevents this effect. Cells. 2022;11(19):3128. 36231090 10.3390/cells11193128 PMC9563448

[50] Shu X, Wei C, Tu WY, et al Negative regulation of TREM2-mediated *C9orf72* poly-GA clearance by the NLRP3 inflammasome. Cell Rep. 2023;42(2):112133. 36800288 10.1016/j.celrep.2023.112133

[51] Deora V, Lee JD, Albornoz EA, et al The microglial NLRP3 inflammasome is activated by amyotrophic lateral sclerosis proteins. Glia. 2020;68(2):407–421. 31596526 10.1002/glia.23728

[52] Debye B, Schmülling L, Zhou L, Rune G, Beyer C, Johann S. Neurodegeneration and NLRP3 inflammasome expression in the anterior thalamus of SOD1(G93A) ALS mice. Brain Pathol. 2018;28(1):14–27. 27880990 10.1111/bpa.12467 PMC8028558

[53] Johann S, Heitzer M, Kanagaratnam M, et al NLRP3 inflammasome is expressed by astrocytes in the SOD1 mouse model of ALS and in human sporadic ALS patients. Glia. 2015;63(12):2260–2273. 26200799 10.1002/glia.22891

[54] Bellezza I, Grottelli S, Costanzi E, et al Peroxynitrite activates the NLRP3 inflammasome cascade in SOD1(G93A) mouse model of amyotrophic lateral sclerosis. Mol Neurobiol. 2018;55(3):2350–2361. 28357805 10.1007/s12035-017-0502-x

[55] Gugliandolo A, Giacoppo S, Bramanti P, Mazzon E. NLRP3 inflammasome activation in a transgenic amyotrophic lateral sclerosis model. Inflammation. 2018;41(1):93–103. 28936769 10.1007/s10753-017-0667-5

[56] Neumann M . Molecular neuropathology of TDP-43 proteinopathies. Int J Mol Sci. 2009;10(1):232–246. 19333444 10.3390/ijms10010232 PMC2662455

[57] Zhao W, Beers DR, Bell S, et al TDP-43 activates microglia through NF-κB and NLRP3 inflammasome. Exp Neurol. 2015;273:24–35. 26222336 10.1016/j.expneurol.2015.07.019

[58] Jiang S, Maphis NM, Binder J, et al Proteopathic tau primes and activates interleukin-1β via myeloid-cell-specific MyD88- and NLRP3-ASC-inflammasome pathway. Cell Rep. 2021;36(12):109720. 34551296 10.1016/j.celrep.2021.109720 PMC8491766

[59] Stancu IC, Cremers N, Vanrusselt H, et al Aggregated Tau activates NLRP3–ASC inflammasome exacerbating exogenously seeded and non-exogenously seeded Tau pathology in vivo. Acta Neuropathol. 2019;137(4):599–617. 30721409 10.1007/s00401-018-01957-y PMC6426830

[60] Ising C, Venegas C, Zhang S, et al NLRP3 inflammasome activation drives tau pathology. Nature. 2019;575(7784):669. 31748742 10.1038/s41586-019-1769-z PMC7324015

[61] Hull C, Dekeryte R, Buchanan H, et al NLRP3 inflammasome inhibition with MCC950 improves insulin sensitivity and inflammation in a mouse model of frontotemporal dementia. Neuropharmacology. 2020;180:108305. 32931815 10.1016/j.neuropharm.2020.108305

