# Supplementary material for: C9orf72 dipeptides activate the NLRP3 inflammasome
Source: Brain Commun. 2024 Aug 20;6(5):fcae282. doi: 10.1093/braincomms/fcae282 (PMC11369816; doi:10.1093/braincomms/fcae282)

## Slide 1
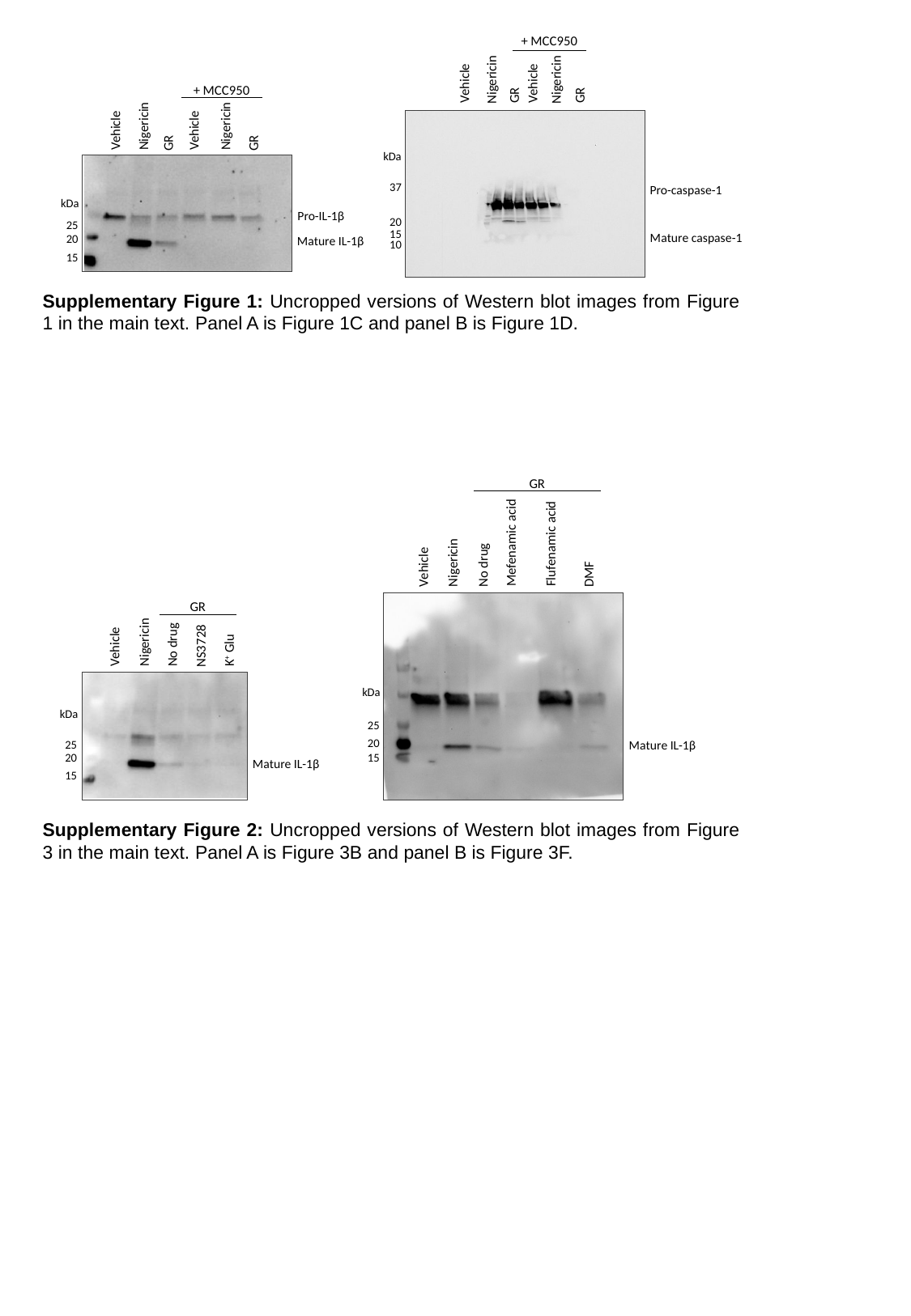

+ MCC950
Nigericin
Nigericin
Vehicle
Vehicle
GR
GR
kDa
37
Pro-caspase-1
20
15
Mature caspase-1
10
+ MCC950
Nigericin
Nigericin
Vehicle
Vehicle
GR
GR
kDa
Pro-IL-1β
25
20
Mature IL-1β
15
Supplementary Figure 1: Uncropped versions of Western blot images from Figure 1 in the main text. Panel A is Figure 1C and panel B is Figure 1D.
GR
Mefenamic acid
Flufenamic acid
Nigericin
No drug
Vehicle
DMF
kDa
25
20
Mature IL-1β
15
GR
Nigericin
No drug
NS3728
Vehicle
K+ Glu
kDa
25
20
Mature IL-1β
15
Supplementary Figure 2: Uncropped versions of Western blot images from Figure 3 in the main text. Panel A is Figure 3B and panel B is Figure 3F.

## Slide 2
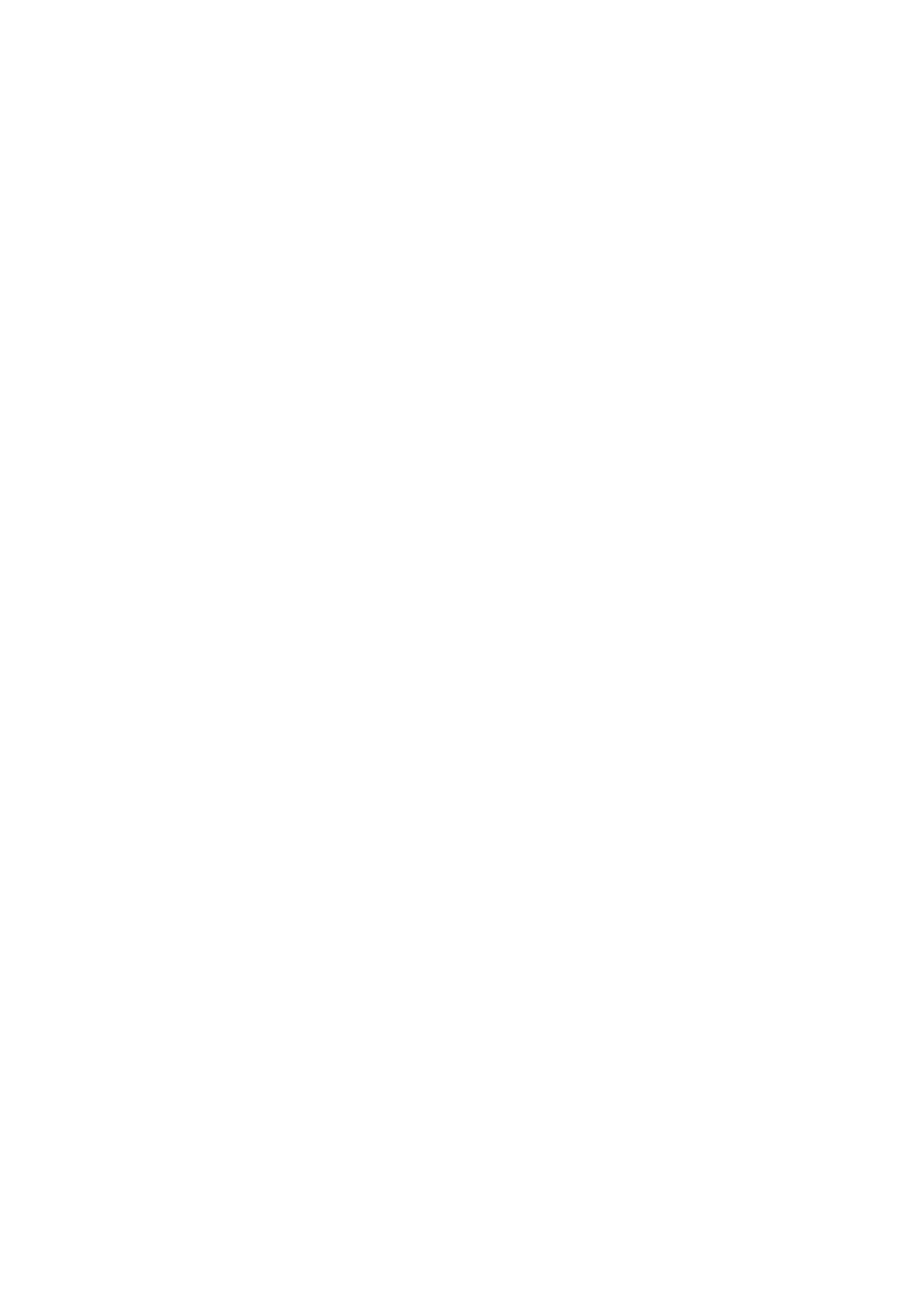

Supplement: fcae282_Supplementary_Data [file fcae282_supplementary_data.zip › Supplementary_material.pptx]
